# Antibody response to BK polyomavirus as a prognostic biomarker and potential therapeutic target in prostate cancer

**DOI:** 10.18632/oncotarget.3363

**Published:** 2015-01-31

**Authors:** Xavier Etienne Keller, Piotr Kardas, Claudio Acevedo, Giovanni Sais, Cédric Poyet, Irina Banzola, Ashkan Mortezavi, Burkhardt Seifert, Tullio Sulser, Hans H. Hirsch, Maurizio Provenzano

**Affiliations:** ^1^ Oncology Research Unit, Division of Urology and Division of Surgical Research, University Hospital Zurich, CH-8091 Zurich, Switzerland; ^2^ Transplantation and Clinical Virology, Department Biomedicine (Haus Petersplatz), University of Basel, CH-4003 Basel, Switzerland; ^3^ Epidemiology, Biostatistics and Prevention Institute, University of Zurich, CH-8001 Zurich, Switzerland; ^4^ Infectious Diseases & Hospital Epidemiology, University Hospital Basel, CH-4031 Basel, Switzerland

**Keywords:** BK polyomavirus, prostate cancer, prognosis, antibody response, biochemical recurrence

## Abstract

Infectious agents, including the BK polyomavirus (BKPyV), have been proposed as important inflammatory pathogens in prostate cancer. Here, we evaluated whether the preoperative antibody response to BKPyV large T antigen (LTag) and viral capsid protein 1 (VP1) was associated with the risk of biochemical recurrence in 226 patients undergoing radical prostatectomy for primary prostate cancer. Essentially, the multivariate Cox regression analysis revealed that preoperative seropositivity to BKPyV LTag significantly reduced the risk of biochemical recurrence, independently of established predictors of biochemical recurrence such as tumor stage, Gleason score and surgical margin status. The predictive accuracy of the regression model was denotatively increased by the inclusion of the BKPyV LTag serostatus. In contrast, the VP1 serostatus was of no prognostic value. Finally, the BKPyV LTag serostatus was associated with a peculiar cytokine gene expression profile upon assessment of the cellular immune response elicited by LTag. Taken together, our findings suggest that the BKPyV LTag serology may serve as a prognostic factor in prostate cancer. If validated in additional studies, this biomarker may allow for better treatment decisions after radical prostatectomy. Finally, the favorable outcome of LTag seropositive patients may provide a potential opportunity for novel therapeutic approaches targeting a viral antigen.

## INTRODUCTION

Prostate cancer is the most commonly diagnosed solid malignancy among males in western countries (233'000 estimated new cases in 2014 in the US) [1]. Currently established prognostic clinicopathologic parameters insufficiently differentiate between indolent and aggressive prostate cancer, leading to both over- and undertreatment of this disease [2]. Hence, an increasing amount of gene and protein biomarkers are considered for a more individualized risk stratification, but their implementation in routine clinical practice is still under evaluation [3, 4]. Therefore, a novel biomarker addressing an established model of prostate carcinogenesis might serve as a clinically valuable prognostic factor and could uncover a therapeutic target allowing for an effective tumor control.

Chronic inflammation is considered to be one of the main factors contributing to prostate cancer development [5]. Among other inflammatory-related infectious agents, the human BK polyomavirus (BKPyV) has been proposed as an important pathogen involved in the transition of normal prostate glands towards overt prostate cancer [6]. This virus is ubiquitous in the human population and establishes a persistent asymptomatic infection in the urinary tract [7]. BKPyV replication is orchestrated by the viral regulatory protein large T antigen (LTag) and leads to lysis of permissive cells with the release of viral progeny. Such a lytic productive infection elicits an antibody response to the viral capsid protein 1 (VP1) in most healthy humans [8]. In the case of an abortive infection – where LTag expression is uncoupled from VP1 expression – LTag still exerts a substantial host cell stimulation that may transform into oncogenic activity by binding products of tumor suppressor genes, such as p53 and retinoblastoma proteins (pRB) [9]. In the context of the latter event, an antibody response against LTag can be elicited.

Investigating the antibody response to both BKPyV VP1 and LTag in prostate cancer patients could clarify the role of these antigens as key targets of cancer immune surveillance. In this study, we evaluated biochemical recurrence free survival (RFS) – as defined by the rate of patients without prostate specific antigen (PSA) relapse over time after radical prostatectomy (RP) – according to the preoperative BKPyV serostatus of primary prostate cancer patients. In addition, we verified the specificity of the antibody response to BKPyV LTag by analyzing the cell-mediated immune response to this antigen in selected patients. The final aim was to evaluate BKPyV serology as a potential prognostic biomarker for prostate cancer recurrence.

## RESULTS

All of the 206 patients available for analysis had an adenocarcinoma of the prostate and their clinicopathologic characteristics are summarized in Supplementary Table S1. In two patients, no prostate cancer was found in the prostatectomy specimen (pTx), although positive prostate biopsies were confirmed.

**Figure 1:**
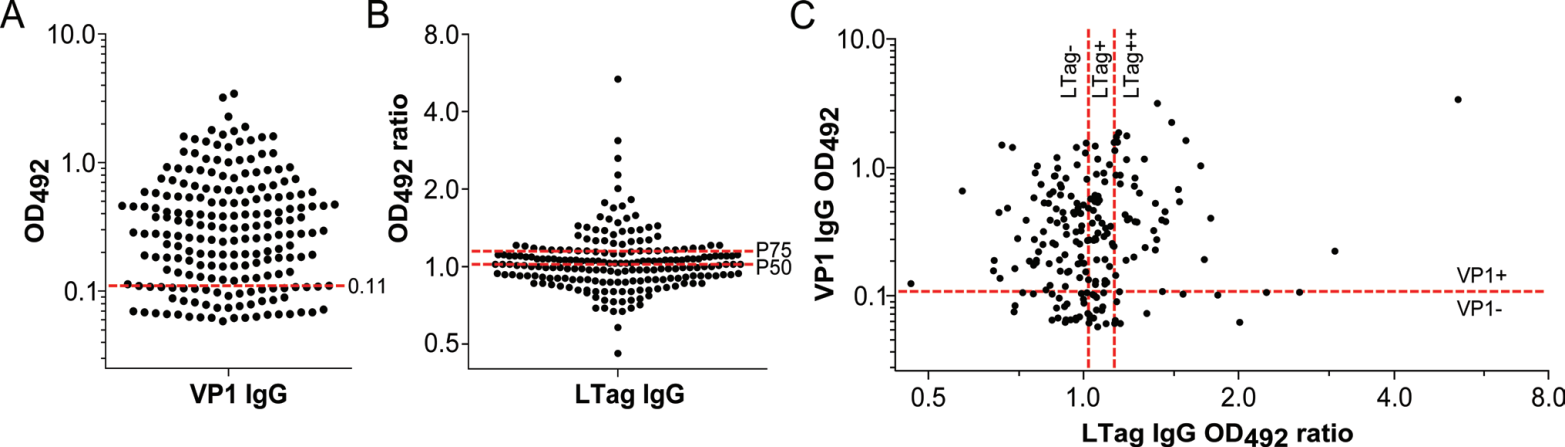
Antibody activity against BKPyV VP1 and LTag in patients who underwent RP for primary prostate cancer (n = 206) (A) Column scatter graph of the VP1 IgG optical density values at 492 nm (OD_492_). The established cutoff (OD_492_ = 0.11) defining VP1 seropositivity (VP1+) was marked by a red dashed line. (B) Column scatter graph of the LTag IgG OD_492_ ratio values. Following cutoffs defined LTag seropositivity: overall 50^th^ percentile (P50) OD_492_ ratio (1.0226) for borderline seropositive patients (LTag+) and overall 75^th^ percentile (P75) OD_492_ ratio (1.1478) for strongly seropositive patients (LTag++) (red dashed lines). (C) Scatter plot correlating VP1 and LTag IgG activity. Quadrants were generated according to the selected VP1 and LTag cutoffs (red dashed lines).

The immunoglobulin G (IgG) serological assay results and the selected cutoffs differentiating VP1 seronegative from (VP1-) seropositive patients (VP1+) as well as the cutoffs differentiating LTag seronegative (LTag−), borderline LTag seropositive (LTag+) and strongly LTag seropositive patients (LTag++) are reported in Fig. 1. The LTag-cutoffs were defined as the 50^th^ and the 75^th^ percentile of all the LTag IgG ratio values (1.0226 and 1.1478, respectively), corresponding to the closest quartile to a ratio of 1 and to the next highest quartile, respectively. Accordingly, 159/206 (77%) patients were VP1+ (Fig. 1A), 52/206 (25%) LTag+ and 51/206 (25%) LTag++ (Fig. 1B). No association was found between VP1 and LTag serostatus (Table 1). When only VP1 and LTag seropositive patients were considered (*n* = 78), a trend for a moderate correlation between VP1 and LTag activity was found (Spearman's rho = 0.22, p = 0.05) (Fig. 1C).

**Table 1:** Distribution of BKPyV VP1 by LTag serostatus

	LTag serostatus			
Characteristic	LTag− (*n*=103)	LTag+ (*n*=52)	LTag++ (*n*=51)	p
VP1 serostatus				
VP1−	22 (21%)	13 (25%)	12 (24%)	0.87^a^
VP1+	81 (79%)	39 (75%)	39 (77%)	

a Two-sided Pearson Chi-square test.

The contingency table analysis did not provide any evidence for an association between the BKPyV serostatus and the clinicopathologic parameters (age at operation, PSA at diagnosis, tumor stage, nodal status, Gleason score and surgical margin status), other than a trend for a low proportion of Gleason score 5-6 in the LTag+ group and for a low proportion of Gleason score 8-9 in the LTag++ group (Table 2).

**Table 2:** Distribution of patient clinicopathologic characteristics by BKPyV serostatus

	BKPyV serostatus						
Characteristic	VP1− (*n*=47)	VP1+ (*n*=159)	p	LTag− (*n*=103)	LTag+ (*n*=52)	LTag++ (*n*=51)	p
Age at operation							
<63 y	19 (40%)	74 (46%)	0.46^a^	47 (46%)	25 (48%)	21 (41%)	0.77^a^
≥63 y	28 (60%)	85 (54%)		56 (54%)	27 (52%)	30 (59%)	
PSA at diagnosis							
<10 ng/mL	41 (87%)	126 (79%)	0.22^a^	84 (82%)	41 (79%)	42 (82%)	0.89^a^
≥10 ng/mL	6 (13%)	33 (21%)		19 (18%)	11 (21%)	9 (18%)	
Tumor stage							
<pT3	36 (77%)	124 (78%)	0.84^a^	78 (76%)	39 (75%)	43 (84%)	0.42^a^
≥pT3	11 (23%)	35 (22%)		25 (24%)	13 (25%)	8 (16%)	
Nodal status							
pN0	26 (55%)	86 (54%)	0.82^b^	53 (51%)	32 (61%)	27 (53%)	0.50^b^
pN1	2 (4%)	5 (3%)		3 (3%)	3 (6%)	1 (2%)	
Unknown	19 (40%)	68 (43%)		47 (46%)	17 (33%)	23 (45%)	
Gleason score							
5-6	11 (23%)	27 (17%)	0.59^a^	21 (20%)	5 (10%)	12 (24%)	0.08^a^
7	30 (64%)	112 (70%)		68 (66%)	37 (71%)	37 (73%)	
8-9	6 (13%)	20 (13%)		14 (14%)	10 (19%)	2 (4%)	
Surgical margin							
R0	35 (75%)	108 (68%)	0.39^a^	70 (68%)	35 (67%)	38 (75%)	0.66^a^
R1	12 (25%)	51 (32%)		33 (32%)	17 (33%)	13 (25%)	

a Two-sided Pearson Chi-square test.

b Two-sided Fisher's exact test.

To search for an association between BKPyV serostatus and the clinical course of the disease after RP, we performed survival analyses based on time to biochemical recurrence (BR). To ensure the quality of our follow-up data, we analyzed the estimates of RFS before and after stratification for established predictors of BR. Over a median follow-up of 48 months (range = 13–70), a BR was documented in 43 patients. The overall estimated RFS was 87% (95% confidence interval [CI], 83–92) at 24 months and 77% (95% CI, 71–84) at 48 months after surgery (Supplementary Fig. S1). High tumor stage, high Gleason score and positive surgical margins were associated with significantly lower estimates of RFS (p < 0.001, p < 0.001 and p = 0.001, respectively), whereas no significant difference in the estimates of RFS was found for PSA stratification (p = 0.31) (Supplementary Fig. S2).

**Figure 2:**
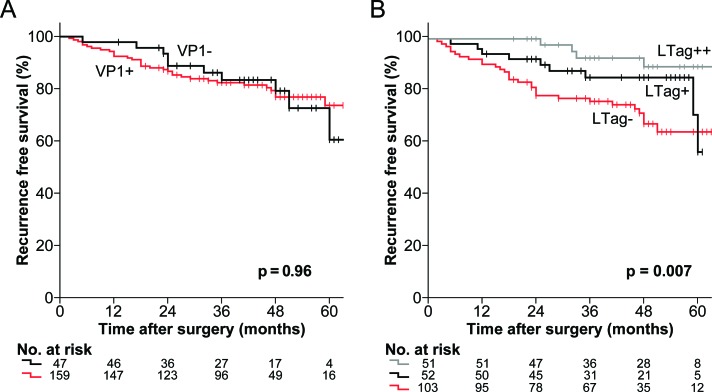
Kaplan-Meier estimates of RFS by BKPyV serostatus in patients who underwent RP for primary prostate cancer (A) Estimates of RFS stratified by the established cutoff for VP1 seropositivity (OD_492_ = 0.11) (black line = VP1- and red line = VP1+). (B) Estimates of RFS stratified by the 50^th^ and the 75^th^ percentile LTag IgG OD_492_ ratio (1.0226 and 1.1478, respectively) (grey line = LTag++, black line = LTag+ and red line = LTag−). All p values were two-sided log-rank tests.

For VP1 serostatus stratification, no significant difference in the estimates of RFS was found (p = 0.96) (Fig. 2A). Higher IgG activity cutoffs, differentiating patients with a stronger antibody response to VP1, did not reveal any significant differences either (Supplementary Fig. S3). By contrast, LTag+ and LTag++ patients showed significantly higher estimates of RFS than LTag− patients (p = 0.007) (Fig. 2B). Notably, the estimated RFS at 48 months was 89% (95% CI, 79–99) for LTag++ patients, 85% (95% CI, 75–95) for LTag+ patients and 68% (95% CI, 57–78) for LTag− patients.

**Figure 3:**
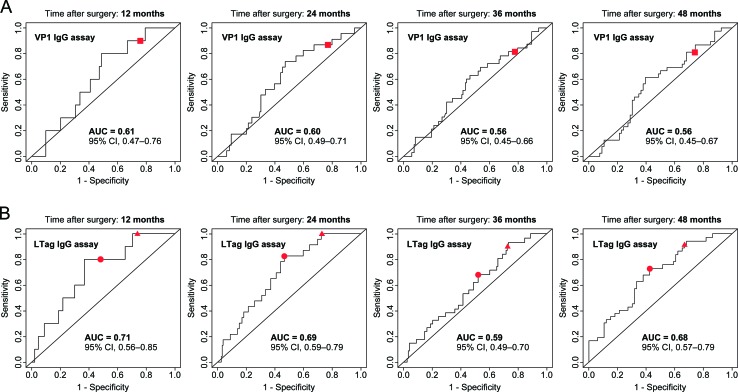
Evaluation of the predictive accuracy of the BKPyV serological assays based on estimates of RFS in patients who underwent RP for primary prostate cancer (A) Time-dependent ROC curves for the VP1 IgG assay prediction of BR at 12, 24, 36 and 48 months after surgery. Curves were calculated based on continuous VP1 IgG activity levels, with the established cutoff (OD_492_ = 0.11) symbolized by a red square. (B) Time-dependent ROC curves for the LTag IgG assay prediction of BR at 12, 24, 36 and 48 months after surgery. Curves were calculated based on continuous LTag IgG activity levels, with the overall 50^th^ percentile LTag IgG OD_492_ ratio (1.0226) and the overall 75^th^ percentile LTag IgG OD_492_ ratio (1.1478) cutoff symbolized by a red circle and a red triangle, respectively.

To further evaluate the predictive accuracy of the BKPyV VP1 and LTag serological assays for BR, time-dependent receiver operating characteristic (ROC) curve analyses were performed based on RFS at 12, 24, 36 and 48 months after surgery. For the VP1 IgG assay, the 95% CI of the area under the curve (AUC) persistently included the 0.50 limit, thus revealing no evidence for a prognostic potential (Fig. 3A). For the LTag IgG assay, the AUC reached 0.68 (95% CI, 0.57–0.79) at 48 months (Fig. 3B). The predictive accuracy of established predictors of BR yielded similar results; at 48 months, the AUC was 0.68 (95% CI, 0.57–0.78) for tumor stage, 0.67 (95% CI, 0.58-0.76) for Gleason score, 0.63 (95% CI, 0.54–0.73) for surgical margin status and 0.57 (95% CI, 46–69) for PSA level at diagnosis (Supplementary Fig. S4).

In the multivariate analysis, LTag+ and LTag++ patients maintained a significantly lower risk of BR than LTag− patients, independent of established predictors of this event (tumor stage, Gleason score and surgical margin status) (Table 3). The predictive accuracy of the Cox regression model – as quantified by the Harrell's concordance index – could be increased from 0.76 (95% CI, 0.68 – 0.83) to 0.79 (95% CI, 0.71 – 0.86) before and after inclusion of the BKPyV serostatus variable, respectively. PSA and VP1 serostatus did not reach the entry level criterion for the forward-stepwise Cox regression model and these parameters were therefore not included in the multivariate analysis.

**Table 3:** Multivariate Cox regression analysis of the risk of BR for BKPyV serostatus stratification, adjusting for established predictors of BR

Variable	Parameter			HR	(95% CI)	p^a^
BKPyV serostatus	LTag+	vs.	LTag−	0.44	(0.21 – 0.93)	0.032
	LTag++	vs.	LTag−	0.25	(0.09 – 0.71)	0.009
Tumor stage	pT≥3	vs.	pT<3	3.5	(1.8 – 6.8)	<0.001
Gleason score	≥7b	vs.	≤7a	2.1	(1.1 – 3.7)	0.034
Surgical margin	R1	vs.	R0	2.4	(1.1 – 4.0)	0.018

HR = hazard ratio

a All *P* values were two-sided.

**Figure 4:**
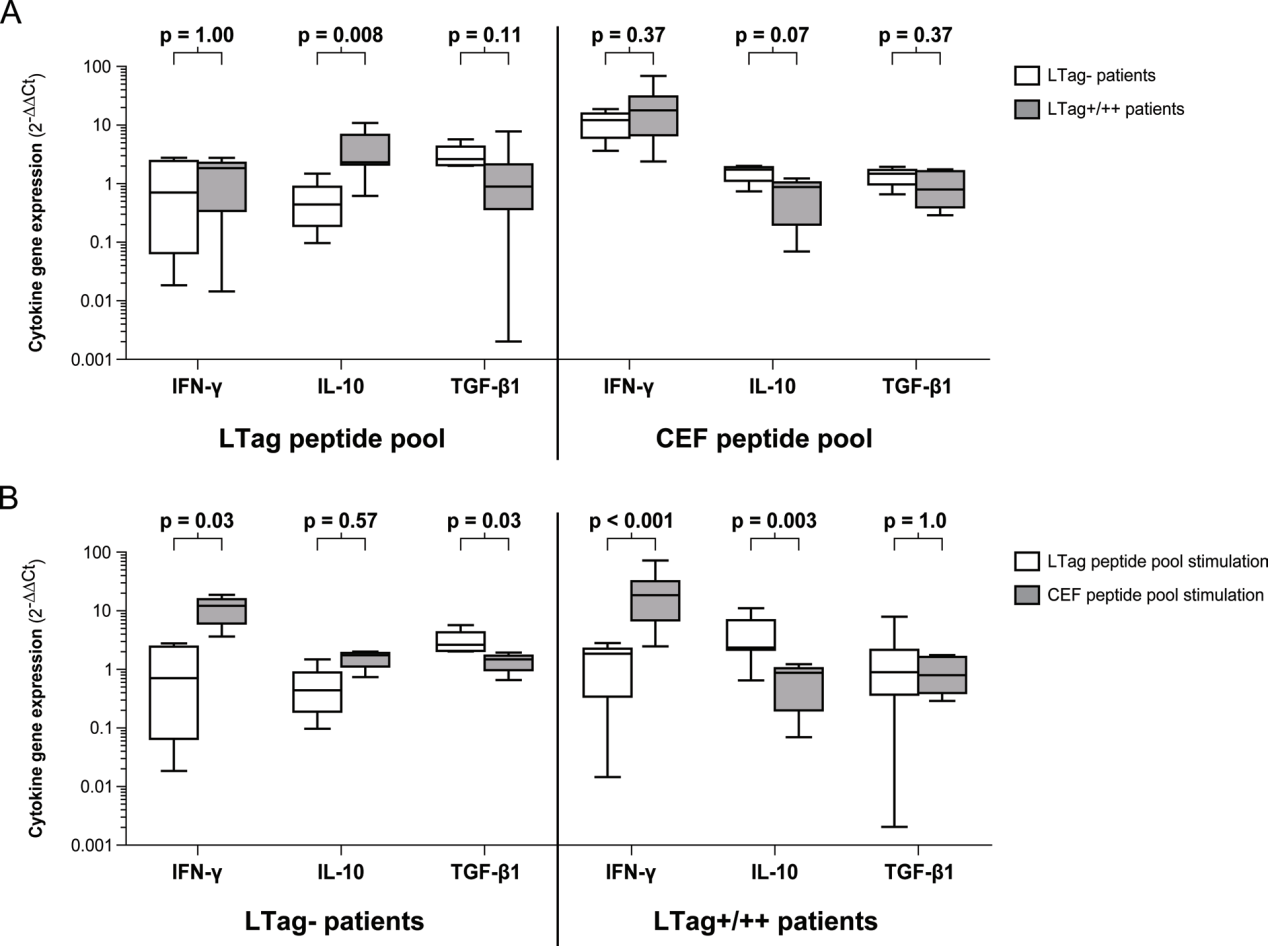
Cytokine gene expression upon peptide pool stimulation of PBMCs from 12 selected age-, stage- and grade-matched prostate cancer patients (A) Box-and-whiskers plots comparing the IFN-γ, IL-10 and TGF-β1 gene expression levels between LTag seronegative patients (LTag−) and LTag borderline seropositive and strongly seropositive patients (LTag+/++) upon either LTag or CEF peptide pool stimulation. (B) Box-and-whiskers plots comparing the IFN-γ, IL-10 and TGF-β1 gene expression levels upon either LTag or CEF peptide pool stimulation in the group of LTag seronegative patients (LTag−) and in the group of LTag borderline seropositive and strongly seropositive patients (LTag+/++). Distributions of cytokine gene expression levels variables were compared by Mann-Whitney U tests. All p values were two-sided.

The favorable prognosis of LTag seropositive patients prompted us to verify a finding that we previously reported, namely that the LTag serostatus was associated with a peculiar cellular immune response profile upon LTag antigen stimulation. For this purpose, we selected 12 age-, stage- and grade-matched patients with the aim of having 4 patients per LTag serostatus group (i.e. LTag−, LTag+ and LTag++). Patients' peripheral blood mononuclear cells (PBMCs) were *ex vivo* stimulated either with a BKPyV LTag peptide pool or with a CEF (cytomegalovirus, Epstein-Barr virus and influenza virus) peptide pool for the analysis of the gene expression of three specific cytokines: interferon gamma (IFN-γ), interleukin 10 (IL-10) and transforming growth factor β1 (TGF-β1). Comparisons were made between the group of LTag seronegative patients (LTag−, n = 4) and the group of LTag seropositive patients (LTag+/++, n = 8) (Fig. 4A). After LTag peptide pool stimulation, no evidence for differing IFN-γ gene expression levels was found between the LTag− group and the LTag+/++ group (p = 1.00). Notably, the LTag+/++ group showed significantly higher IL-10 gene expression levels than the LTag− group (p *=* 0.008), while higher TGF-β1 gene expression levels were observed in the LTag− group (p = 0.11) (Fig. 4A). In contrast, the CEF peptide pool stimulation showed no evidence for differing cytokine gene expression levels upon BKPyV LTag serostatus stratification (Fig. 4A).

To verify whether the observed *ex vivo* cellular immune responses of these 12 patients were indeed specific to the BKPyV LTag, we compared the cytokine gene expression patterns induced by either the LTag or the CEF peptide pool. In both the LTag− and the LTag+/++ group, the CEF peptide pool stimulation induced considerably higher IFN-γ gene expression levels than the LTag peptide pool stimulation (p = 0.03 and p < 0.001, respectively) (Fig. 4B). Furthermore, after CEF peptide pool stimulation of the PBMCs from LTag+/++ patients, the IL-10 gene expression levels remained significantly lower than after LTag peptide pool stimulation (p = 0.003) (Fig. 4B). This confirms that the IL-10 elevation observed in LTag+/++ patients is indeed specific to the LTag peptide pool stimulation. Finally, in the LTag− group, a trend towards a higher TGF-β1 gene expression upon LTag− peptide pool stimulation was found (p = 0.03) (Fig. 4B).

## DISCUSSION

Cumulative evidence for a co-factorial role of the BKPyV in prostate carcinogenesis has emerged from prostate tissue analyses and immune response characterization studies in prostate cancer patients [10-12]. To our knowledge, this is the first study addressing the prognostic significance of the antibody response to BKPyV in prostate cancer patients.

In comparison to Merkel cell carcinoma patients, where high Merkel cell polyomavirus VP1 IgG levels were associated with a better progression-free survival [13], no evidence for an association between the BKPyV VP1 serostatus and the risk of prostate cancer recurrence was provided in this study. This finding underlines the notion that a productive BKPyV infection – leading to a strong VP1 IgG activity – is unlikely to reflect the oncogenic potential of the BKPyV, as permissive cells will be lysed upon release of the viral progeny [14].

Rather, the novelty of our investigation is the finding that an antibody response to the viral regulatory protein LTag is associated with the clinical course of prostate cancer patients after RP. Because the LTag may lead to the oncogenic transformation of BKPyV infected glandular prostate cells, an increased antibody activity against this oncogenic protein would be expected in cases with high risk of BR. Indeed, in cervical cancer patients, antibodies against human papillomavirus proteins E6 and E7, whose functions are similar to LTag, are markers of worse prognosis [15]. Differently, in this study, increasing estimates of RFS were found over increasing levels of preoperative BKPyV LTag IgG activity. Particularly, about 89% of all strongly LTag seropositive patients were free of BR at 48 months after RP, whereas only 68% of all LTag seronegative patients were BR-free at 48 months. This finding was highlighted in the multivariate analysis adjusting for the usually available patient clinicopathologic characteristics, which indicated that the risk of BR was lowered by at least 29% in strongly LTag seropositive patients and that even borderline seropositivity for BKPyV LTag led to a risk reduction of at least 7%. Of note, the addition of the BKPyV serostatus variable in the multivariate Cox regression model resulted in a denotative increase of the predictive accuracy of the model, thus suggesting the preoperative BKPyV serostatus as a potentially valuable biomarker for the prediction of BR after RP.

The almost equal distribution of the VP1 serostatus over the three levels of the LTag serostatus reveals that more than half of all VP1 seronegative patients must have had LTag antigens exposed to their immune system in the absence of a VP1 antigenic stimulation. This exemplifies that the uncoupled continuous expression of LTag with the lacking VP1 expression, typically occurring in abortively infected cells, can be effectively targeted by the immune system. However, to date, it is unknown whether the prostate is the main site for the antigenic exposition of BKPyV LTag to the immune system and the detection of LTag in areas of overt prostate cancer remains elusive and needs to be standardized [16]. Several studies regarding the association between BKPyV and prostate cancer led to the common conclusion that the virus exerts its oncogenic activity at early stages of cancer development [10]. Particularly, LTag has frequently been detected in areas of proliferative inflammation atrophy (PIA), which is considered a precursor lesion leading to prostatic intraepithelial neoplasia and overt prostate cancer [17]. The absence of any association between the BKPyV serostatus and the usual patient clinicopathologic characteristics, such as tumor stage or Gleason score, underlines the concept that BKPyV has an etiological role only in precancerous and early stage cancer lesions surrounding tumor areas.

The cytoplasmic localization of LTag in complex with wild type p53 in abortively infected cells, as seen at the PIA level [10, 18], makes this antigen a suitable target for the generation of memory CD8^+^ and CD4^+^ T cells. Indeed, reactivation of HLA class I effector memory CD8^+^ T cells and class II polyfunctional CD4^+^ T cells with dual T-helper and T-cytotoxic properties was observed in BKPyV seropositive healthy subjects experiencing smoldering infections [19-21]. Furthermore, peculiar cellular immune response profile was recalled by the LTag stimulation of PBMCs in BKPyV seropositive prostate cancer patients [11].

Correspondingly, we assessed the cytokine gene expression of PBMCs upon *ex vivo* LTag peptide pool stimulation and could verify our previous findings. Notably, we were able to recall a strong IL-10 gene expression in LTag seropositive patients and additionally characterized a trend for an increased TGF-β1 gene expression in LTag seronegative patients. Both IL-10 and TGF-β1 are characteristic of type 1 T regulatory cells and are frequently reported as immunoregulatory cytokines impairing cancer immune surveillance [22-24]. Yet, IL-10 has also been found to induce effective antitumor immune responses in various studies [25]. The latter might explain why the LTag peptide pool stimulation would recall the expression of IL-10 in the favorable prognosis group of LTag seropositive patients. Hence, a favorable microenvironment allowing for effective immune-mediated tumor surveillance would explain the long-lasting absence of BR in most patients showing strong LTag seropositivity enrolled in this study.

We could verify the PBMCs' ability to recall a strong IFN-γ immune response upon stimulation with the immunogenic CEF peptide pool. In addition, no evidence for a significant increase in IL-10 or TGF-β1 cytokine gene expression levels was found after CEF peptide pool stimulation. This was in agreement with our expectations, as the CEF peptide pool is used for its ability to recall effector immune responses and stimulates CD8+ T cells to preferentially produce IFN-γ. Hereby, we not only attributed the specificity of our findings to the BKPyV, but also verified that the individual variability of the LTag−specific antibody activity was not due to individual differences of age-related decline of immune functions. The relative immunological integrity of the evaluated cohort is further attested by the overall VP1 seroprevalence of 77%, which is in agreement with previous age-stratified seroepidemiological studies on polyomaviruses in blood donors [26, 27].

On account of these findings, the elicitation of a functional immune response against the BKPyV LTag by therapeutic means might increase the amount of specific prostate-infiltrating T lymphocytes with effector functions or redirect existing effector T cells with an exhausted phenotype, thereby inducing a favorable local tumor immune microenvironment allowing for immune-mediated tumor surveillance and consequently lower the risk of BR after RP [28].

Although more than 200 prostate cancer patients were tested for BKPyV serostatus using subtype 1/b1 for VP1-based virus-like particles (VLP) generation – which is the most common BKPyV subtype among Caucasians [29] – the present study is not devoid of limitations. Particularly, patients' ethnicity and antigenically distinct BKPyV serotypes might represent possible confounding factors of our estimates of RFS [30, 31]. Moreover, a potential LTag−specific antibody cross-reactivity between the BKPyV and other human polyomaviruses – such as the endemic JC Polyomavirus (JCPyV) – might have contributed to the LTag IgG ratio values [32]. However, so far, no rationale for a role of polyomavirures other than the BKPyV has been demonstrated in prostate cancer [33]. Notably, in our prior investigations, we could not find any JCPyV-specific LTag sequences at indicative copy numbers in prostatic tissues, whereas BKPyV-specific LTag sequences were readily detectable in 42% of the PCa tissue specimens and 32% of the benign prostatic hyperplasia tissues [11]. Based on the findings reported by Βodaghi et al., the rise of IgG activity levels against BKPyV-specific LTag subdomain 1 (LTD1) is preceded by an extensive BKPyV viremia in immunocompromised kidney transplanted patient [34]. Accordingly, we consider it most plausible to assume that the IgG activity observed in our investigation reflects BKPyV LTag exposure to the immune system. Additional studies on human polyomaviruses' LTag IgG seroprevalence and its distribution over age groups in healthy populations would be needed for clarification.

Another limitation of our study is the absence of evidence for an association between the PSA levels at diagnosis and the risk of BR, the former being an established risk-predictor. This might be related to the relatively short follow-up or to the intrinsic imprecisions of PSA measurements, which were not standardized. Nevertheless, our findings are based on a representative prostate cancer patient cohort undergoing RP, as reflected by the significant and independent performance of other established predictors of BR, such as tumor stage, Gleason score and surgical margin status. Hence, the reported results are intriguing enough to justify further characterization of the role of BKPyV in prostate cancer.

In conclusion, our investigation provides an unprecedented epidemiological evidence for an association between antibody responses to BKPyV LTag and the clinical course of prostate cancer. Particularly, we reveal that the preoperative LTag serology may be a valuable biomarker for the prediction of favorable prostate cancer prognosis after RP. If validated in additional studies, this biomarker may allow for better postoperative treatment decisions. Finally, this study suggests a promising opportunity for novel therapeutic approaches targeting a virus-related oncogenic antigen in early prostate cancer lesions.

## METHODS

### Study population

We evaluated a case series of 226 patients undergoing RP for primary prostate cancer in our institution between October 2007 and November 2011. A prospective observational tumor marker study design was used, according to Simon et al. [35]. Whole-mount prostatectomy sections were analyzed by the Institute of Surgical Pathology of the University Hospital of Zurich. Tumor grades and stages were assigned according to the International Society of Urological Pathology (ISUP 2005) [36] and Union for International Cancer Control (UICC 2002 and 2009) [37, 38], respectively. When no prostate cancer was found in the prostatectomy specimen (pTx), Gleason score at biopsy was reported. None of the patients had received neoadjuvant androgen-deprivation therapy.

The primary endpoint was time from surgery to BR, with censoring of patients without BR at the date of last follow-up. For this purpose, patients were routinely assessed by PSA measurements at 6 weeks, 3, 6 and 12 months postoperatively and annually thereafter. BR was defined as a rise in PSA levels ≥0.1 ng/mL after surgery. Serum PSA level testing was not standardized, since many patients were initially diagnosed and followed up in referral centers. Overall, 20 patients unfit for survival analyses were excluded for the following reasons: i) postoperative PSA measurements not available (*n* = 4); ii) postoperative PSA nadir (PSA level drop <0.1 ng/mL) not reached (*n* = 13); iii) administration of a second-line therapy at PSA levels <0.1 ng/mL (*n* = 3). Local ethics committee approval and written informed consent from all patients were provided. This study was conducted following the Reporting Recommendations for Tumor Marker Prognostic Studies (REMARK) guidelines [39].

### Blood specimen collection and handling

Blood specimens were collected before surgery. Serum tubes were centrifuged at 1300 x g for 10 min at room temperature. After centrifugation, serum specimens (1 mL aliquots) were frozen within 2 hours after collection and stored at −80°C until use. PBMCs were isolated from venous blood by Ficoll-Hypaque density gradient centrifugation (Histopaque, Sigma-Aldrich, St. Louis, MO, USA) and stored in liquid nitrogen until use.

### Serological analysis

Serological assays were performed blinded to the patients' characteristics and outcome. Antibody responses to BKPyV were measured by enzyme immunoassays (EIA) based on two BKPyV antigens: the VP1 in the form of VLP and the N-terminal 133-aa LTag subdomain 1 (LTD1) conjugated with glutathione-S-transferase (GST-LTD1 fusion protein). These two antigens were produced as described previously [34]. Briefly, VLP were isolated from *Spodoptera frugiperda* Sf9 cells (American Type Culture Collection [ATCC], Manassas, VA, USA) infected with recombinant baculovirus encoding BKPyV subtype 1/b1 VP1 (Bac-to-Bac baculovirus expression system, Invitrogen, Carlsbad, CA, USA). Infected Sf9 cells were disrupted by sonication and glass mortar and pestle treatment. VLP were purified from cellular lysate by CsCl gradient. The 3-dimensional conformation of VLP was confirmed by transmission electron microscopy. For LTD1 expression, the recombinant baculovirus transfer plasmid was ligated with a pFastBac1-GST vector (Invitrogen, Carlsbad, CA, USA) allowing for single-step GST-fusion protein purification by glutathione-affinity chromatography (Glutathione Sepharose 4B, GE Healthcare, Piscataway, NJ, USA). Purified non-VLP expressing cell lysates and purified GST were used as negative controls.

After coating EIA wells with 100 ng of VLP or 25 ng of GST-LTD1 fusion proteins, BKPyV-specific VP1 or LTag IgG activity, respectively, was measured as an optical density value at 492 nm (OD_492_) with a serum dilution of 1:400 using an automated plate reader (Tecan Group Ltd., Männedorf, Switzerland), as described previously [34]. For each antigen, all serum samples were analyzed on the same day. For VP1 IgG, we adopted the cutoff OD_492_ = 0.11 after subtraction of purified non-VLP expressing cell lysates OD_492_ value (negative control) to differentiate seronegative (VP1-) from seropositive patients (VP1+), as established elsewhere [8, 34, 40, 41]. For LTag IgG, the ratio of the GST-LTD1 IgG OD_492_ value divided by its respective affinity-purified GST IgG OD_492_ value (negative control) was calculated for each patient. This allowed for normalization of inter-individual variations of unspecific IgG binding to GST. Ideally, an LTag IgG ratio of >1 would define a patient as seropositive. Therefore – and since a standard cutoff for the LTag IgG activity had not been defined at the time of this study execution – we considered the quartile of all LTag IgG OD_492_ ratio values closest to a ratio of 1 to differentiate between seronegative (LTag−) and borderline seropositive patients (LTag+) and the next highest quartile to discriminate strongly seropositive (LTag++) patients. This allowed for specific comparisons between seronegative (LTag−), borderline seropositive (LTag+) and strongly seropositive patients (LTag++).

### *Ex vivo* induction and cytokine gene expression by qRT-PCR

After thawing, PBMCs were stimulated with either a BKPyV LTag peptide pool, a positive control CEF (cytomegalovirus, Epstein-Barr virus and influenza virus) peptide pool, or a negative control HIV peptide pool (JPT Peptide Technology, Berlin, Germany), as previously described [11]. Quantitative gene amplification was performed by using a Corbett Rotor-Gene 3000 Instrument (Corbett Life Science, Sydney, Australia) with a TaqMan Universal PCR master mix reagents kit and “on demand” sets of primers and probes for cytokine gene expression of IFN-γ, IL-10 and TGF-β1 (Applied Biosystems, Rotkreuz, Switzerland), as previously described [42]. The negative control was used to compute cytokine gene expression fold changes. Finally, the normalized data were analyzed by the 2^−ΔΔCt^ method using β-Actin as an endogenous reference gene [43].

### Statistical analysis

Categorical variables were evaluated by contingency table analyses and Pearson Chi-square tests or Fisher's exact tests, as appropriate. Mann-Whitney U tests were run to determine differences between continuous independent variables. Association between continuous variables was assessed by Spearman's rank correlation coefficient. Estimates of RFS were calculated with the Kaplan-Meier method and were compared with the log-rank test. Based on these estimates of RFS, the predictive accuracy of the considered BKPyV seromarkers was evaluated by time-dependent ROC curves. A forward-stepwise multivariate Cox regression analysis (entry level at 0.05) was modeled to evaluate the antibody response to BKPyV as a predictor of BR, adjusting for established predictors of BR. Proportional hazard assumption was assessed for each variable with the plot of a log-negative-log survival distribution and by the plot of Schoenfeld's residuals over time. The predictive accuracy of the Cox regression model was estimated using the Harrell's concordance index. Owing to multiple comparisons, the two-sided significance level was set at 0.01, exception made for the multivariate Cox regression analysis. The latter represents a single test that includes confounding covariates, justifying a significance level of 0.05.

Column scatter graphs were generated with GraphPad Prism 5.04 (GraphPad Software, La Jolla CA, USA). Time-dependent ROC curves were estimated with the timeROC v0.2 package for R [44]. The Harrell's concordance index was based on forward-stepwise multivariate Cox regression analysis results using Stata 13.1 (Stata Corp., College Station, TX, USA). Analyses of all other variables were performed with IBM SPSS Statistics 22.0 (IBM Corp., Armond, NY, USA).

## SUPPLEMENTARY MATERIAL, FIGURES AND TABLE


