# Supplementary material for: Antibody response to BK polyomavirus as a prognostic biomarker and potential therapeutic target in prostate cancer
Source: Oncotarget. 2015 Jan 31;6(8):6459–69. doi: 10.18632/oncotarget.3363 (PMC4467449; doi:10.18632/oncotarget.3363)
Supplement: Supplementary file 1 [file oncotarget-06-6459-s001.pdf]

Antibody response to BK polyomavirus as a prognostic biomarker and potential therapeutic target in prostate cancer

Supplementary Material

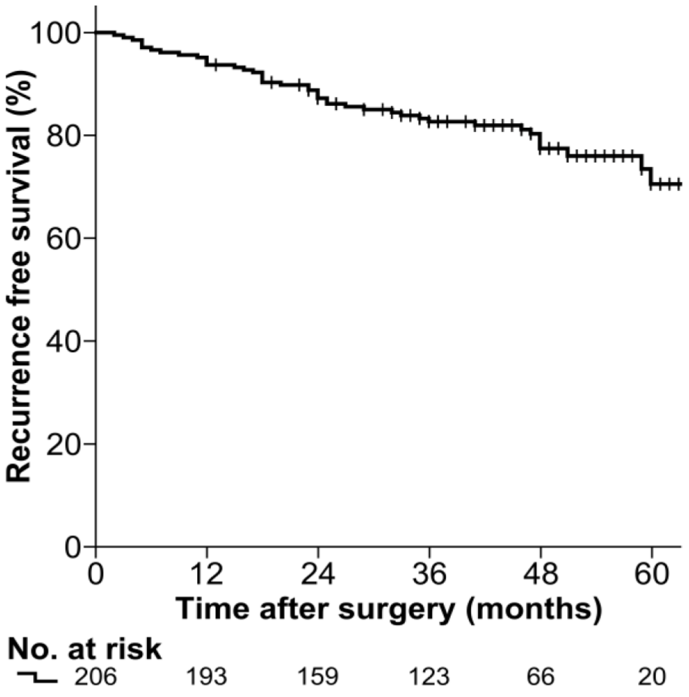

Supplementary Figure S1: Kaplan-Meier estimates of the overall RFS in patients who underwent RP for primary prostate cancer.

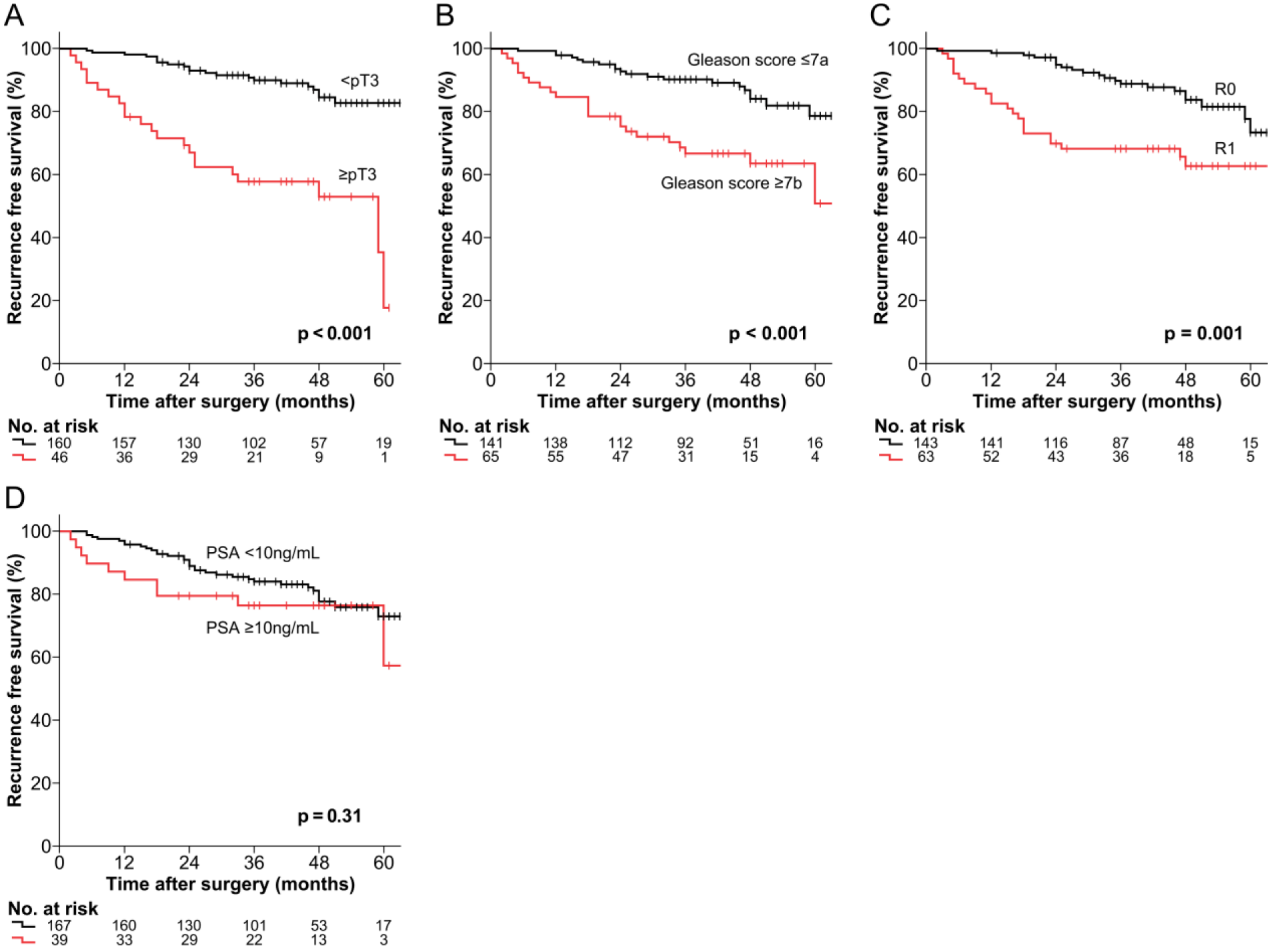

Supplementary Figure S2: Kaplan-Meier estimates of RFS in patients who underwent RP for primary prostate cancer. (A-D) Patients were dichotomized by tumor stage (black line <pT3 vs. red line ≥pT3) **(A)**, Gleason score (black line ≤7a vs. red line ≥7b) **(B)**, surgical margin status (black line = R0 vs. red line = R1) **(C)** and PSA level at diagnosis (black line <10ng/mL vs. red line ≥10ng/mL) **(D)**. All p values were two-sided log-rank tests.

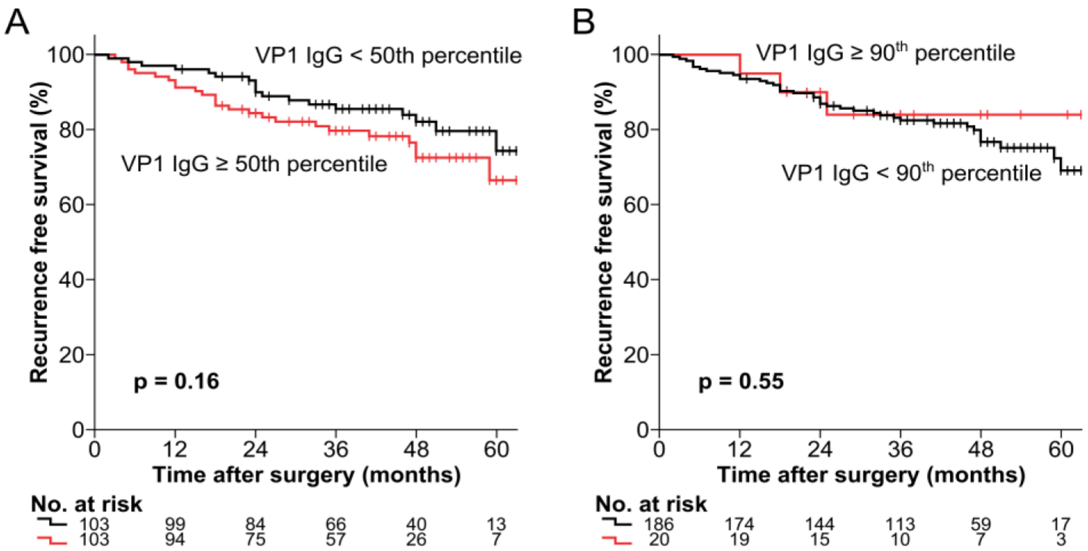

Supplementary Figure S3. Kaplan-Meier estimates of RFS in patients who underwent RP for primary prostate cancer. **(A)** Estimates of RFS dichotomized by the median VP1 antibody activity (OD_{492} = 0.28) (black line <50th percentile vs. red line ≥50th percentile). **(B)** Estimates of RFS dichotomized by the 90th percentile VP1 antibody activity (OD_{492} = 1.10) (black line <90th percentile vs. red line ≥90th percentile).

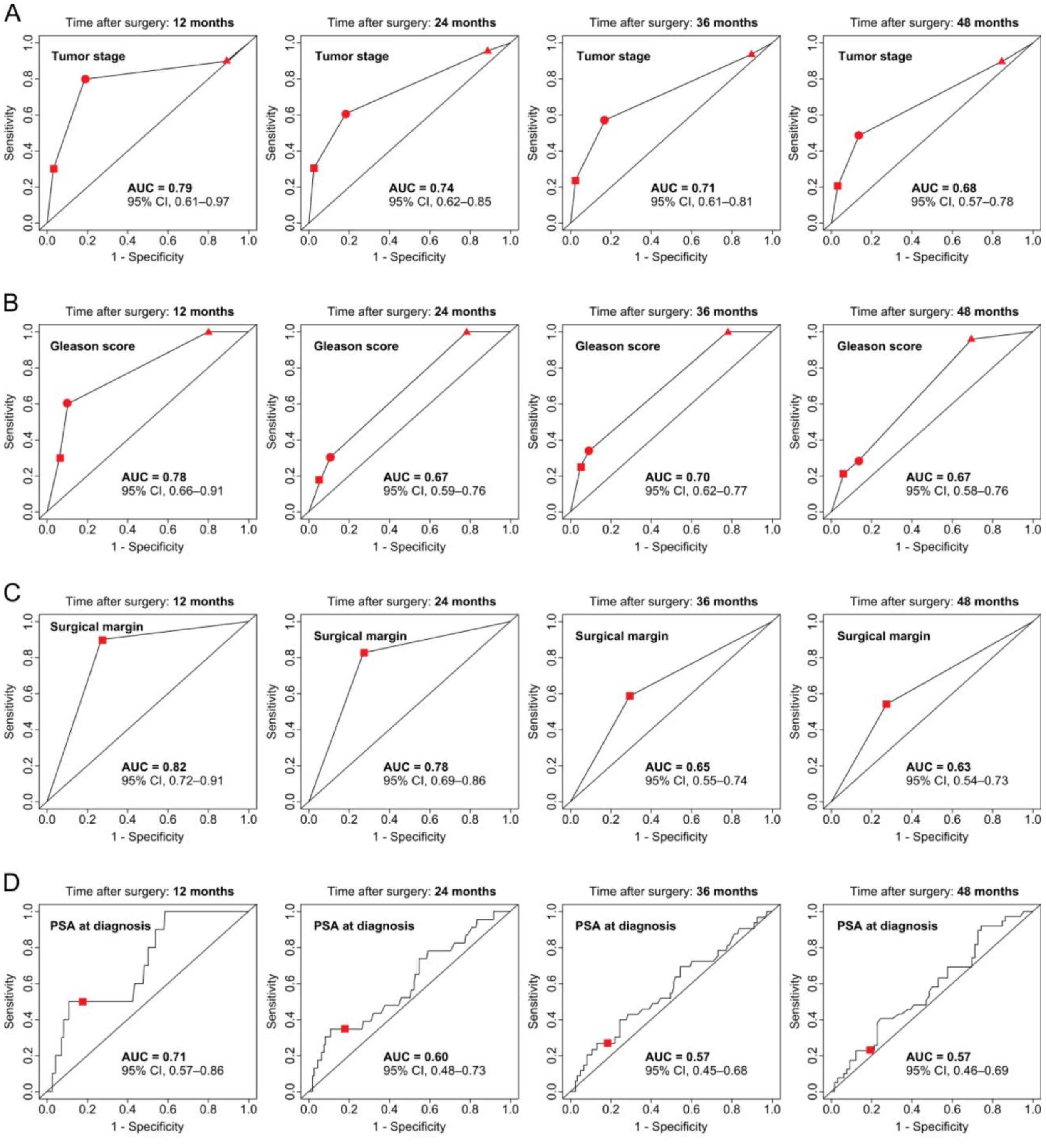

Supplementary Figure S4. Evaluation of the predictive accuracy of established predictors of BR based on RFS in patients who underwent RP for primary prostate cancer. (A) Time-dependent ROC curves for tumor stage prediction of BR at 12, 24, 36 and 48 months after surgery. Curves were calculated based on the distinction between following stages: pT4 vs. pT3 (red square), pT3 vs. pT2c (red circle) and pT2c vs. pTx or pT2a/b (red triangle). **(B)** Time-dependent ROC curves for Gleason score prediction of BR at 12, 24, 36 and 48 months after surgery. Curves were calculated based on the distinction between

following scores: 9 vs. 8 (red square), 8 vs. 7 (red circle) and 7 vs. 6 (red triangle). **(C)** Time-dependent ROC curves for surgical margin status prediction of BR at 12, 24, 36 and 48 months after surgery. Curves were calculated based on the distinction between R1 vs. R0 (red square). **(D)** Time-dependent ROC curves for the prediction of BR by PSA levels at diagnosis at 12, 24, 36 and 48 months after surgery. Curves were calculated based on continuous PSA levels, with the cutoff at 10ng/ml symbolized by a red square.

Table S1. Patient clinicopathologic characteristics of the study population

**Characteristic**		
Age at operation, y.		
Median (range)	63	(43–74)
PSA at diagnosis, ng/mL		
Median (range)	6.2	(1.2–43)
Tumor stage, no. (%)		
pTx	2	(1)
pT2a	17	(8)
pT2b	3	(2)
pT2c	138	(67)
pT3a	35	(17)
pT3b	11	(5)
Nodal status, no. (%)		
pN0	112	(55)
pN1	7	(3)
Unknown	87	(42)
Gleason score[a], no. (%)		
3+2 = 5	1	(1)
3+3 = 6	37	(18)
3+4 = 7a	103	(50)
4+3 = 7b	39	(19)
4+4 = 8	11	(5)
4+5 or 5+4 = 9	15	(7)
Surgical margin, no. (%)		
R0	143	(69)
R1	63	(31)

[a]For two patients staged pTx, the Gleason score at biopsy was reported.
